# Promising Molecular Architectures for Two-Photon Probes in the Diagnosis of α-Synuclein Aggregates

**DOI:** 10.3390/molecules29122817

**Published:** 2024-06-13

**Authors:** Stefania Porcu, Riccardo Corpino, Carlo Maria Carbonaro, Pier Carlo Ricci, Attilio Vittorio Vargiu, Anna Laura Sanna, Giuseppe Sforazzini, Daniele Chiriu

**Affiliations:** 1 Department of Physics, University of Cagliari, Cittadella Universitaria, SP n°8, 09042 Monserrato, CA, Italy; stefania.porcu@dsf.unica.it (S.P.); riccardo.corpino@dsf.unica.it (R.C.); cm.carbonaro@dsf.unica.it (C.M.C.); carlo.ricci@dsf.unica.it (P.C.R.); vargiu@dsf.unica.it (A.V.V.); 2 Department of Chemistry and Hearth Science, University of Cagliari, Cittadella Universitaria, SP n°8, 09042 Monserrato, CA, Italy; sannaannalaura@gmail.com

**Keywords:** two-photon-excited (TPE) probes, molecular docking, in vivo probing, early PD detection, targeted intervention strategies

## Abstract

The abnormal deposition of protein in the brain is the central factor in neurodegenerative disorders (NDs). These detrimental aggregates, stemming from the misfolding and subsequent irregular aggregation of α-synuclein protein, are primarily accountable for conditions such as Parkinson’s disease, Alzheimer’s disease, and dementia. Two-photon-excited (TPE) probes are a promising tool for the early-stage diagnosis of these pathologies as they provide accurate spatial resolution, minimal intrusion, and the ability for prolonged observation. To identify compounds with the potential to function as diagnostic probes using two-photon techniques, we explore three distinct categories of compounds: Hydroxyl azobenzene (AZO-OH); Dicyano-vinyl bithiophene (DCVBT); and Tetra-amino phthalocyanine (PcZnNH_2_). The molecules were structurally and optically characterized using a multi-technique approach via UV-vis absorption, Raman spectroscopy, three-dimensional fluorescence mapping (PLE), time-resolved photoluminescence (TRPL), and pump and probe measurements. Furthermore, quantum chemical and molecular docking calculations were performed to provide insights into the photophysical properties of the compounds as well as to assess their affinity with the α-synuclein protein. This innovative approach seeks to enhance the accuracy of in vivo probing, contributing to early Parkinson’s disease (PD) detection and ultimately allowing for targeted intervention strategies.

## 1. Introduction

The increase in life expectancy highlights the pressing need for the development of innovative diagnostic tools to detect diseases that are inherently linked to aging, among which the most diffused ones are Alzheimer’s, Parkinson’s (PD), and dementia. The distinguishing features of these neurodegenerative diseases (NDs) comprise the intracellular and extracellular formation of protein aggregates, such as neurofibrillary tangles, amyloid plaques, and Lewy bodies [1,2,3]. The diagnosis of PD is mainly based on clinical observation of motor symptoms that only appear at advanced disease stages, while the measurement of reliable biomarkers for early diagnosis is not yet available. Today’s approach to ND clinical diagnosis relies on the use of radiolabeled agents for positron emission tomography (PET) and single-photon emission Computed Tomography (SPECT) technologies [4,5]. These techniques, however, suffer from extended acquisition time and limited spatial resolution, rendering them inadequate for the precise monitoring of disease progression. Optical imaging-based approaches based on two-photon excitation (TPE), on the other hand, can allow for both high spatial and temporal resolution. To date, numerous TPE probes have been developed for detecting β-amyloid plaques and tau aggregates [6,7,8,9,10,11,12,13]. However, to the best of our knowledge, there remains a lack of dedicated probes for α-synuclein (α-syn) aggregates. The abnormal intraneuronal aggregation of α-syn is a neuropathological trademark of PD. Since oligomerization of α-syn precedes neuronal death, the identification via imaging techniques of specific molecular conformation structures of α-syn or early aggregates may allow a prompt detection of the disease and therefore an intervention aimed at slowing or halting the progression of PD. Recent studies have explored the potential of utilizing two-photon-excited amplified spontaneous emission (ASE) from fluorescent dyes to detect protein aggregates of two model fibril-forming proteins, lysozyme and insulin, which form β-sheet-rich aggregates resembling pathological amyloids [14]. The same TPE ASE principle can be potentially exploited towards the detection of α-syn aggregates. To achieve this objective, it is essential to pinpoint molecular diagnostic agents that demonstrate strong nonlinear optical absorption. More specifically, the ideal candidates for the probing of α-syn aggregates must therefore count for (i) affinity or anti-fibrillogenic properties towards α-syn prefibrillar architectures, (ii) two-photon absorption capability, and (iii) low toxicity. By reviewing the recent literature, we identify three possible classes of compounds that can potentially satisfy these three requirements: azobenzenes, malononitrile derivatives, and phthalocyanines. Azobenzene derivatives have found extensive applications in the investigation of β-sheet-rich aggregates [1,15]. They are known to undergo photon-induced isomerization upon absorption of multiple photons [16]. Similarly, malononitrile derivatives have emerged as effective two-photon probes for monitoring fibrils [17,18], while thiophene derivatives have been used as luminescence probes of α-synuclein assemblies [19]. Finally, tetrapyrrolic macrocycles like phthalocyanines and porphyrins have shown anti-fibrillogenic properties against α-synuclein-based aggregates [20,21,22]. Moreover, these macrocycles are renowned for their capability to undergo multi-photon absorption, a phenomenon frequently used in photodynamic cancer treatments [23]. The distinctive characteristics of these compounds position them as promising candidates for ASE fluorescent dyes targeting α-synuclein aggregates.

In this work, part of an extended project, we aim to evaluate the potential of this class of compounds as two-photon probes for fibril imaging, while also establishing putative structure–property relationships to inform the future development of improved diagnostic probes. We began with α-syn as the target compound, intending to extend the analysis to other molecules in the organism in future works.

## 2. Results and Discussion

**Figure 1:**
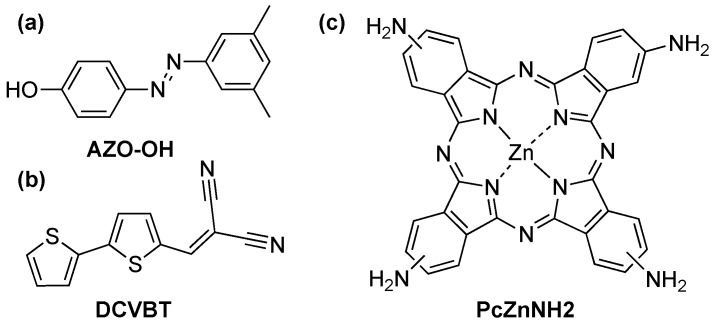
Molecular structure of the compounds investigated in this work: (**a**) Hydroxyl azobenzene (AZO-OH); (**b**) Dicyano-vinyl bithiophene (DCVBT); (**c**) Tetra-amino phthalocyanine (PcZnNH_2_).

The compounds utilized in this study are sketched in Figure 1, providing a visual representation of the chemical entities under investigation.

### 2.1. Raman Characterization

As an initial step in characterizing the structures of the proposed molecules, we conducted Raman spectroscopy analysis, providing Raman vibrational mode assignment for each compound.

**Figure 2:**
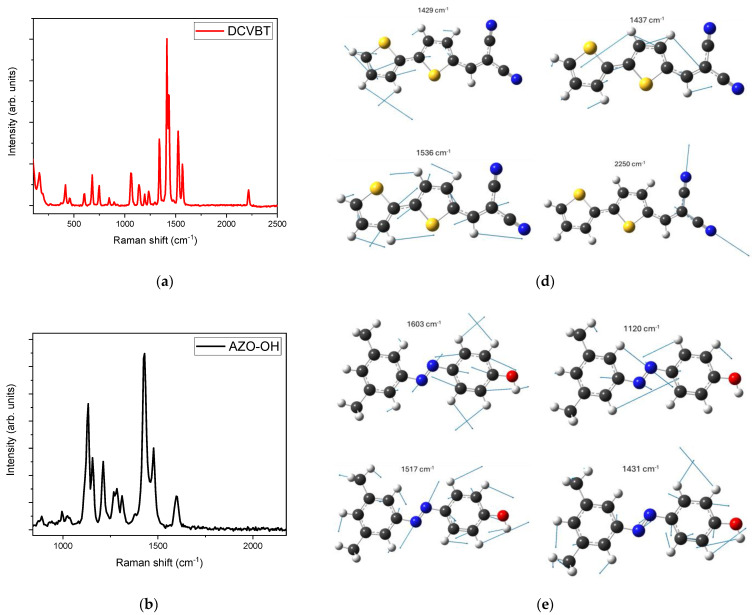

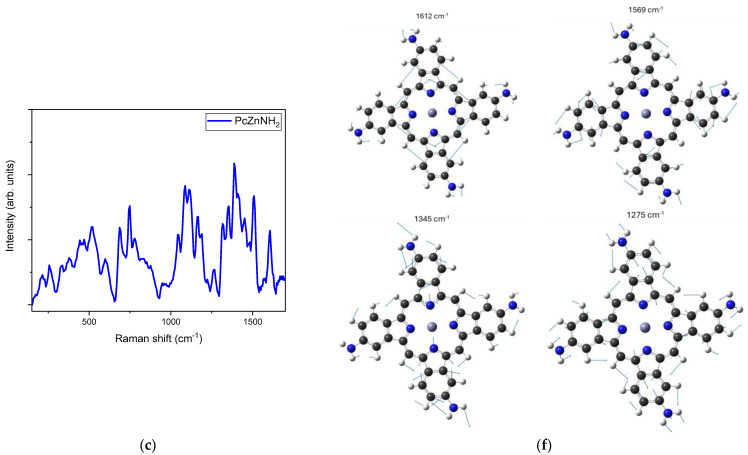
Raman spectra of solid-state molecules excited at 1064 nm: (**a**) DCVBT, (**b**) AZO-OH and (**c**) PCZnNH2. DFT calculation of Raman spectra of which the most representative vibrations are reported: (**d**) DCVBT, (**e**) AZO-OH and (**f**) PCZnNH_2_. In the ball-and-stick representation white sphere = H atom, gray sphere = C atom, red sphere = O atom, blue sphere = N atom, yellow sphere = S atom, and pale purple = Zn atom.

Concerning DCVBT, as shown in Figure 2a, the spectrum presents typical vibrations *ν*(C≡N) of cyano bonds at 2220 cm^−1^, symmetric *ν_s_* and antisymmetric *ν_a_* stretching of double C=C bonds at 1567 and 1525 cm^−1^ and at 1432 and 1414 cm^−1^ for C=C/C-C motions, δ bending for C-C bonds at 1336 cm^−1^, stretching at 1235 and 1193 cm^−1^ for C-C bonds, and internal bending δ vibrations for C-H vinyl at 1140 and 1062 cm^−1^. Below 750 cm^−1^ all the peaks can be assigned to relative motions among macro structures (e.g., thiophenes or cyano groups) [24].

The Raman spectrum of AZO-OH (Figure 2b) exhibits the typical symmetric stretching frequencies (*ν*) of the ring at 1600, 1477 and 1430 cm^−1^; in-plane bending (*β*) for CH (rings) at 1313, 1268, and 1211 cm^−1^; symmetric stretching (*ν*) for CO at 1286, 1133, 1026 and 993 cm^−1^; symmetric stretching (*ν*) for vinylidene CC at 1154 cm^−1^; and, under 800 cm^−1^, typical vibration modes for out-of-plane and in-plane bending for CH (ring), CC, and CO bonds [25].

Finally, in Figure 1c, we report the Raman spectrum of the complex structure of PcZnNH_2_, where the principal vibrations are *A*_1*g*_ modes at 1413, 1317, 1317, 830 and 687 cm^−1^; *E* modes at 1600, 1261, 1044, 781 and 745 cm^−1^; and B2g modes at 1603 and 1115 cm^−1^. Below 600 cm^−1^, all the peaks can be assigned to relative motions among macro structures [26,27]. These assignations are compatible with the DFT calculation of the Raman spectra, the most representative vibrations of which are reported in Figure 2d–f.

### 2.2. Interaction of the Probes with α-syn

In order to investigate the binding propensities of the three molecules towards α-syn, we performed molecular docking calculations using the recent software GNINA 1.0 [28]. When performing ligand–protein docking, it is crucial to take into account protein flexibility [29], particularly in the case of intrinsically disordered proteins (IDPs) such as α-syn, which keep this striking feature within mammalian cells [30]. In fact, Chen et al. [31] recently demonstrated that an α-syn monomer assumes distinct structural subpopulations which can be grouped into eight conformational clusters (hereafter C1 to C8) featuring stable local structures. Furthermore, the authors pinpointed that (i) these monomeric structures could represent structural precursors of the various α-syn pathogenic functions: indeed, dimer, tetramer, and oligomer formation might be stimulated by specific conformational clusters (respectively, C1, C5, and C3 in [31]); (ii) these conformations also include putative membrane-bound conformations (e.g., C6) and (iii) are relatively stable, exhibiting conformational transitions slower than ms. This information was exploited in flexible-ligand ensemble-docking calculations, whereby the three molecules subjected to study were docked on all conformational clusters (see Section 3 for details).

**Figure 3:**
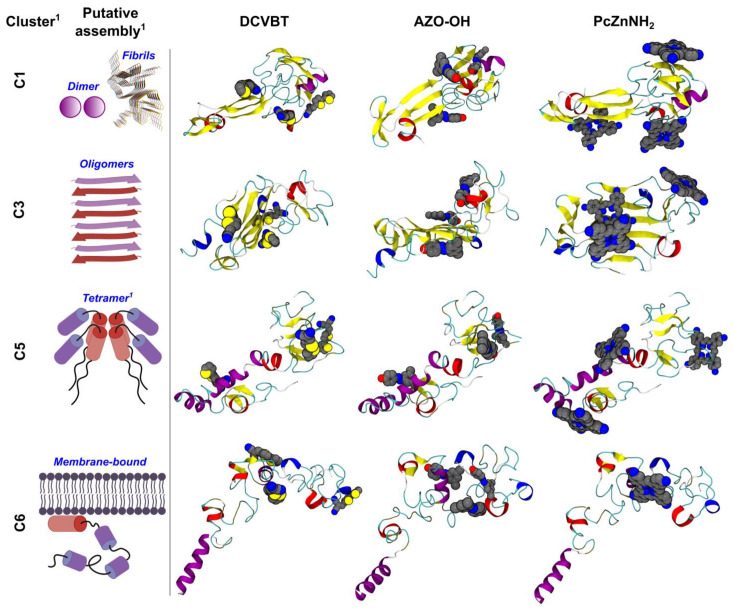
Representative structures of the complexes formed between the three molecules investigated in this work and a-syn, as found in semi-flexible ensemble-docking calculations. **Left** (columns 1 and 2): labeling of the conformational clusters taken from [31] and their putative assembled structures. **Right** (columns 3 to 5): complex representative structures. For each compound, the top three non-overlapping poses are shown as spheres of radii equal to 1.2 (top pose), 1 (second pose), and 0.8 (third pose) in terms of atomic van der Waals radii, colored by atom type (C, N, O, and S in gray, blue, red, and yellow, respectively). The protein is shown using a cartoon representation and colored by secondary structure elements. Created with BioRender.com and VMD 1.9.3 [32].

The results of docking calculations, summarized in Figure 3 and S4 in Supplementary Materials and in Table 1, show that all probes are able to bind with medium (DCVBT and AZO-OH) to high (PcZnNH_2_) affinities to all the conformations of α-syn putatively involved in oligomerization and/or fibrillation processes [31].

### 2.3. Steady-State Absorption and PLE Measurements

**Figure 4:**
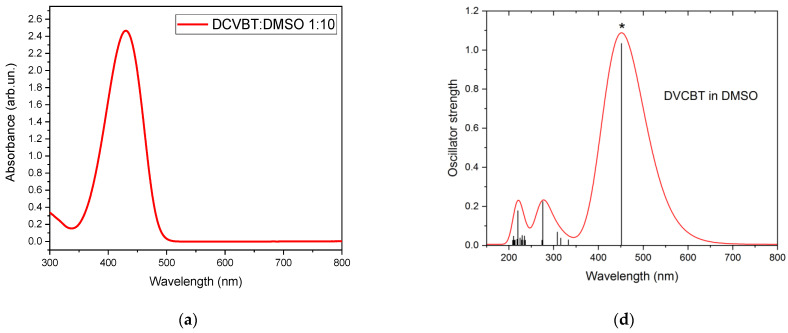

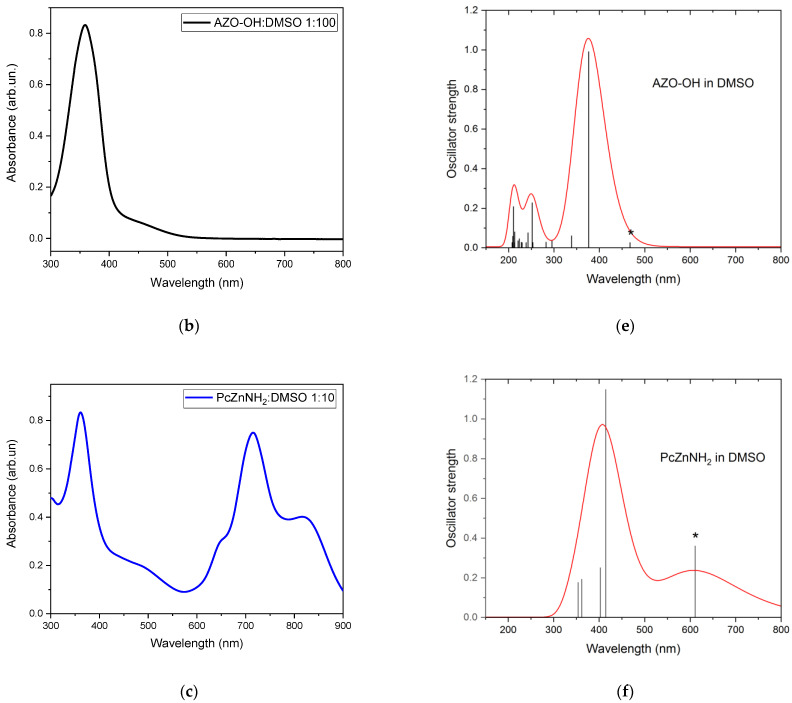
Absorption spectra of DCVBT in DMSO solution 1:10 *w*/*w*% (**a**), AZO-OH in DMSO solution 1:100 *w*/*w*%, (**b**) and PcZnNH_2_ in DMSO solution 1:10 *w*/*w*% (**c**); DFT calculation for the same molecules: DCVBT (**d**), AZO-OH (**e**) and PCZnNH2 (**f**). The * symbol marks the HOMO-LUMO transition. The black lines in d–f represent the oscillator strength.

Steady-state absorption spectra of DMSO-diluted molecules are described in Figure 4. In particular, DCVBT shows a strong absorption centered at 430 nm (Figure 4a), while AZO-OH presents a strong band in the UV region at 370 nm and a shoulder at around 450 nm (Figure 4b). Concerning PCZnNH_2_, the absorption spectrum is more complex than the other molecules because it reveals a multiple structure with bands centered at 360, 500, 650, 700 and 830 nm. Each absorption band is compatible with the DFT calculation of the HOMO-LUMO gap (Highest Occupied and Lowest Unoccupied Molecular Orbital, respectively) and excited states (see Figure 2d–f). Indeed, the H-L gap was estimated at 452 nm, 467 nm and 611 nm for DCVBT, AZO-OH and PCZnNH_2_, respectively, in very good agreement with the experimental findings except for PCZnNH_2_. It should be pointed out that in the latter case, the computed spectrum, despite correctly predicting the optical shape of the first excited states, largely underestimate the gap because of the known reduced ability of the chosen theoretical level in describing the element of the fourth row [33]. The calculated molecular orbitals (MOs) of the three compounds are reported in S3 in Supplementary Materials, showing the typical π–π* transitions involving the aromatic rings of the three molecules.

The absorption properties of DVCBT deserve particular attention, which we selected for a deeper investigation despite it displaying medium affinity for α-synuclein. Indeed, we considered the DVCBT molecule firstly because, in our opinion, its chemical structure and absorption properties may offer unique advantages for our research and, additionally, considering its composition, it might have a more favorable toxicity profile compared to the other molecules under study.

We aimed to highlight its biological compatibility for in vivo experiments. Additionally, we studied the optical properties of DCVBT dispersed in water after preliminary solubilization in a few drops of DMSO, considering that the molecule is completely insoluble in water, a crucial issue for potential bio-applications.

**Figure 5:**
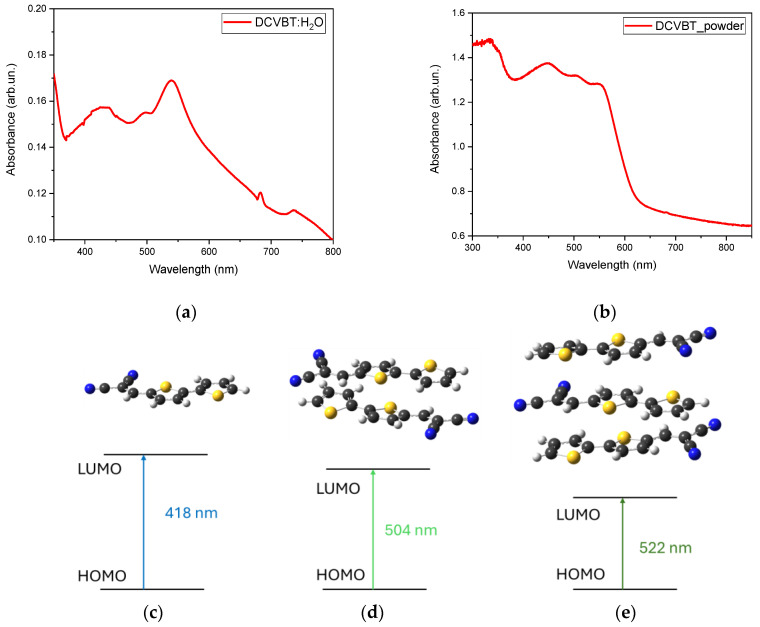
Absorption spectrum of DCVBT in water (**a**) and solid state (**b**); DFT calculation of HOMO LUMO scheme for single molecule (**c**), as well as two (**d**) and three aggregated molecules (**e**). In the ball-and-stick representation, white sphere = H atom, gray sphere = C atom, blue sphere = N atom, and yellow sphere = S atom.

Contrary to the simple spectrum obtained in DMSO solution, in water, there are three bands centered at 430, 500 and 530 nm (Figure 5a). This peculiar behavior can be ascribed to aggregation effects that make the absorption spectrum very similar to that obtained in a solid-state condition (Figure 5b). This assumption is also validated by DFT calculation of progressive aggregation of DCVBT molecules that matches with solid-state absorption when three molecules are grouped together (Figure 5c–e), showing H-L transition at 418 nm, 504 nm, and 522 nm for monomers, dimers, and trimers in vacuum conditions. Further studies will be conducted to enhance the compatibility and solubility of this molecule for biological applications, working on the structural architecture and preserving the optical properties. A possible strategy we plan to explore is to functionalize the molecule with some hydrophilic groups.

For a thorough understanding of the optical properties, we conducted a detailed analysis using photoluminescence excitation mapping. This method allowed us to explore the emission spectra of these molecules extensively, covering a broad range from 250 to 850 nm. By employing this approach, we aimed to capture nuanced details and patterns in the photoluminescence trend, contributing to a comprehensive characterization of their optical properties.

**Figure 6:**
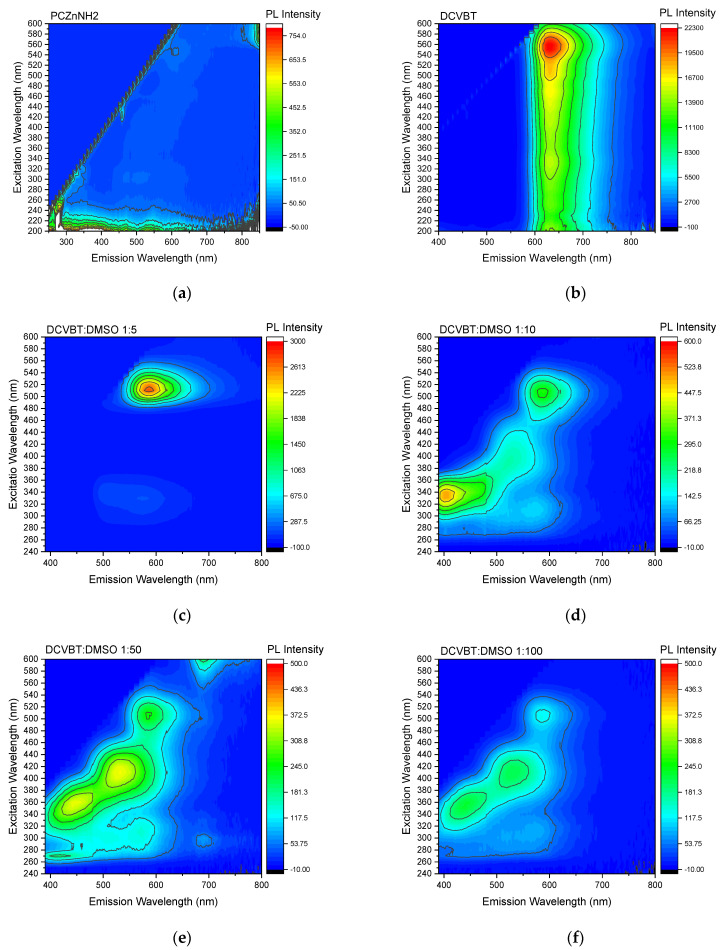
PLE maps of (**a**) solid-state PcZnNH_2_, (**b**) solid-state DCVBT, and DCVBT:DMSO solutions with different concentrations (**c**–**f**).

**Figure 7:**
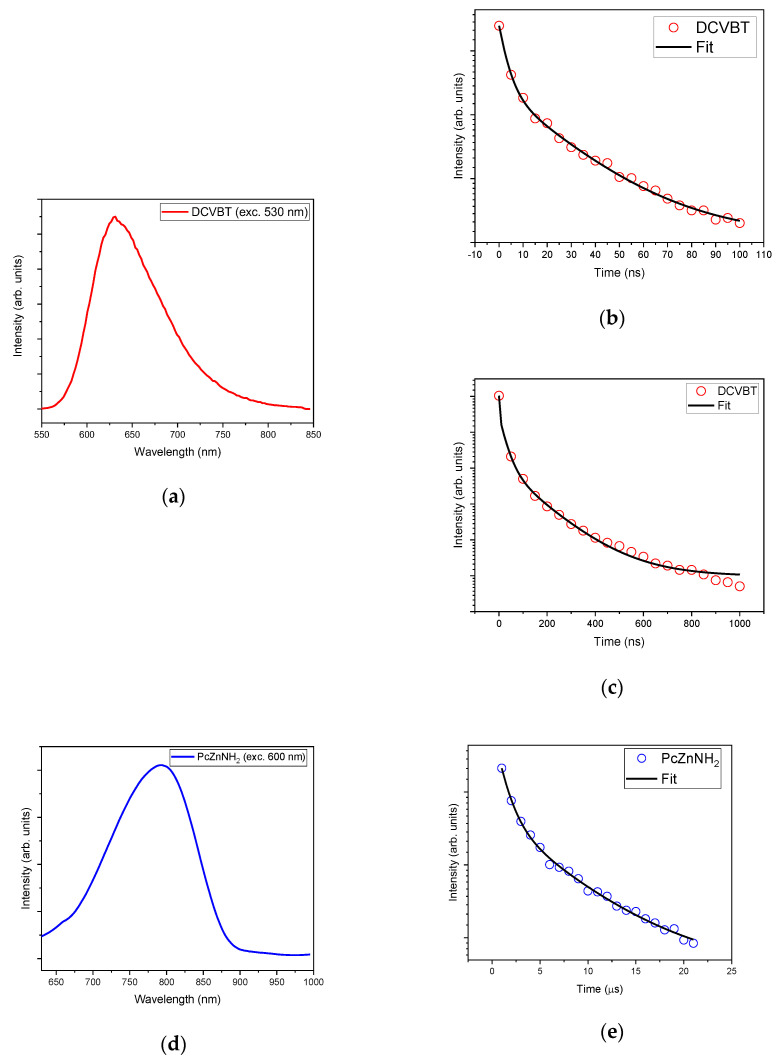
(**a**) Emission of DCVBT molecule and time decays in the range up to 100 ns (**b**) and 1000 ns (**c**); emission of PCZnNH2 (**d**) with relative time decay profile (**e**).

In Figure 6a, we present the excitation maps for the solid-state PcZnNH_2_, exhibiting a low emission peak at 800 nm when excited within the range of 580–600 nm (see Figure 7d for details). In contrast, solid-state DCVBT displays a pronounced emission peak at 630 nm, confirming the presence of three absorption channels for aggregates at 430, 500, and 550 nm (Figure 6b). Conversely, AZO-OH does not exhibit any emission in the same spectral region. To complement our investigation, we also collected photoluminescence excitation (PLE) maps for DCVBT in DMSO solution at various concentrations (Figure 6c–f).

From these photoluminescence excitation (PLE) maps, a notable effect of concentration aggregation becomes apparent. This phenomenon elucidates the transition from the broad, intense emission peak observed at 580 nm in the sample with the highest concentration, primarily excited at 530 nm, to the emergence of emissions at 450 nm and 530 nm at lower concentrations. Notably, for these latter emission bands, the excitation maxima shift towards shorter wavelengths at 400 nm and 360 nm, signifying a change in the underlying photophysical processes. This observation underscores the intricate relationship between concentration, aggregation state, and the resultant optical properties of the studied molecules. For sake of completeness, starting from the emissions obtained in the PLE spectra, we measured the quantum yield of the spontaneous emission of DCVBT and PcZnNH_2_. We found a quantum yield ϕ_DCVBT, DMSO_ = 0.09 ± 0.01 for DCVBT in DMSO, and ϕ_PcZnNH2, DMSO_ = 0.10 ± 0.02 and ϕ_PcZnNH2, Met_ = 0.13 ± 0.02 for PcZnNH_2_ in DMSO and methanol, respectively. For PcZnNH_2_, the results are compatible with reference [34].

### 2.4. Time-Resolved Photoluminescence (TRPL)

Time-resolved photoluminescence (TR-PL) measurements corroborate the aforementioned findings, particularly regarding DCVBT, where three distinct decay times are observed, consistent with the characteristics of the studied aggregates. In Figure 7a, the emission peak at 630 nm is depicted, while Figure 7b,c display exponential fits over different time domains, highlighting a short initial lifetime of approximately 3.5 ns (below the pulse resolution of our setup), a secondary lifetime of 30 ns, and a longer decay time of 163 ns. From this measurement, we suppose that these three characteristic times may be indicative of various aggregation states, aligning with the hypothesis proposed in the previous section.

For comprehensive analysis, TR-PL of PcZnNH_2_ was also considered, confirming emission at 780 nm, with lifetimes of 1.16 μs and 7.98 μs.

### 2.5. Transient Absorption Measurements

**Figure 8:**
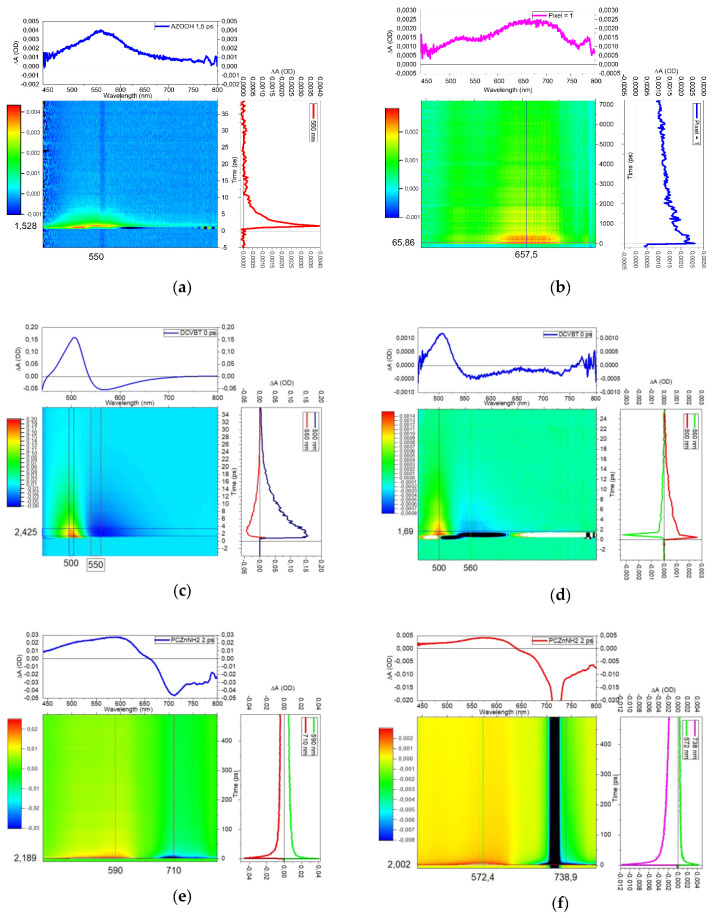
Transient absorption maps with spectral curves and time decays of AZO OH with pump at 400 nm (**a**); AZO-OH with pump at 360 nm (**b**); DCVBT with pump at 420 nm (**c**); DCVBT with pump at 900 nm (**d**); PcZnNH_2_ with pump at 360 nm (**e**); PcZnNH_2_ with pump at 720 nm. Black line is due to the pump laser wavelength (**f**).

To further investigate the optical features of the samples and correlate them to the desired optical properties of these molecules, we performed transient absorption (TA) measurements. Through the study of the positive or negative TA signals, we are able to determine the nature of optical transitions that correspond to Excited-State Absorption (ESA), Ground-State Depletion (GSD) and Stimulated Emission (SE). In Figure 8a,b, we report the TA maps of AZO-OH pumped at 400 nm and 360 nm, respectively. The TA signal was recorded in the 430–800 nm region in the ps time regime. These selected pump values are compatible with the steady-state absorption bands reported in Figure 4.

The first map with excitation at 400 nm presents a short positive signal centered at 550 nm. This optical transition corresponds to an ESA at 2.25 eV beyond the pump photons (3.1 eV). The temporal range of this positive signal is under 10 ps. Under the pump at 360 nm (3.66 eV), the signal remains positive and a new predominant band at 650 nm appears, providing an ESA at 1.91 eV over the first excited state. To finalize the study toward the use of these molecules in two-photon regimes, we moved the pumps to 720 and 900 nm, but no TA signal was detected under the two-photon excitation (2PE) pump for AZO-OH. We conclude that this molecule does not benefit from two-photon absorption properties. On the contrary, the TA results are promising for DCVBT and PcZnNH_2_. Actually, in Figure 8c,d we found that, under pumps at 420 nm (2.95 eV) and 900 nm (1.38 eV), the TA maps present very similar results for DCVBT: a positive signal at 500 nm (ESA at 2.48 eV) and a negative signal corresponding to SE at 560 nm (2.21 eV). To further prove the 2PE properties, we repeated the same measure exciting at 840 nm (1.47 eV). We obtained analogous results of those reported for excitation at 900 nm (not reported for the sake of brevity). The assumption of two-photon absorption is also corroborated by the very narrow time profile displayed in Figure 8d. For PcZnNH_2_, after moving the pump from 360 to 720 nm, we recorded a positive signal at around 570–590 nm (ESA at 2.14 eV) and a negative signal at 750 nm attributed to SE (1.65 eV), as shown in Figure 8e,f. Also in this case, the time profile is characteristic of two-photon absorption. We underline that the negative signal for DCVBT and PcZnNH_2_ could also be assigned to GSD; however, basing our assumption on DFT calculations that do not predict any trapping level near LUMO and the emission features of the two samples, we propose to assign those signals to SE transitions. To give a further indication on the luminescence properties under two-photon absorption, we performed some measurements reported in the S5 of Supporting Information. The results concern the most promising sample, DCVBT (see the right part in S5 of Supporting Information), where the linear fit of the points in a log/log plot (intensity vs. pump fluence) presents a slope of 2 ± 0.1, evidencing the second-order trend associated with this excitation. For DCVBT, the emission by 2PE is clearly demonstrated, while for PcZnNH_2_, we report a reference showing how, in the literature, these properties are widely already known [34]. For completeness, we provide the fluence of the pump during the execution of transient absorption measurements to allow us to hypothesize the optical transitions that are involved in and compatible with two-photon absorption (P = 19 μW, 100 fs pulsed laser, repetition rate 1 kHz, circular spot diameter 0.3 mm).

**Figure 9:**
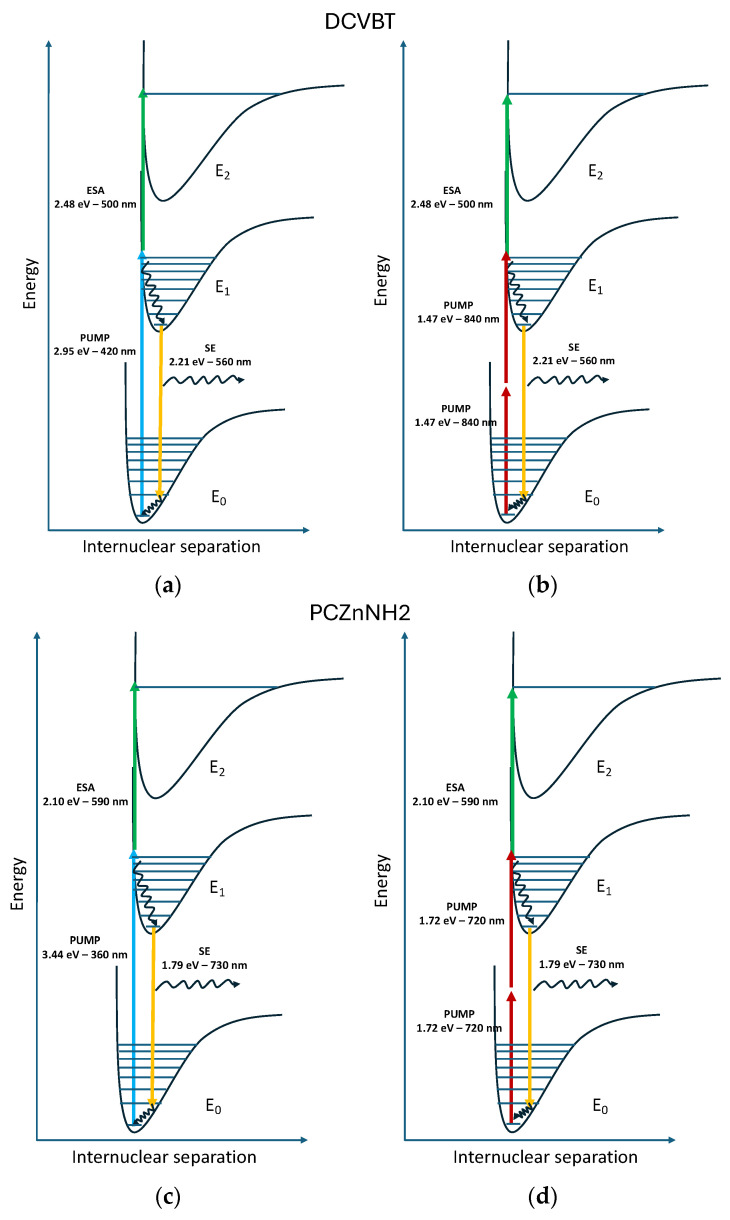
Sketch of energy levels and main transitions determined with optical spectroscopy for DCVBT: (**a**) single-photon pump at 420 nm, (**b**) two-photon pump at 900 nm, (**c**) single-photon pump at 360 nm, and (**d**) two-photon pump at 720 nm.

To sum up the optical features, Figure 9 reports a sketch of energy levels and the main transitions determined with steady-state and transient optical spectroscopy.

By comparing the two molecules, we infer that DCVBT is the most promising one. Indeed, the added value for this molecule is represented by the NIR wavelength of 2PE excitation, 900 nm, that boasts a higher penetration level with respect to the 2PE properties observed for PCZnNH_2_ at 720 nm. Moreover, for DCVBT, the small molecular weight and reduced dimensions make it the best candidate among the analyzed triplet.

## 3. Materials and Methods

### 3.1. Synthesis of Studied Molecules

All solvents and reagents were used as commercially supplied, without further purification. All the solvents (dichloromethane, ethyl acetate, petroleum ether 40–60 °C, ethanol) were purchased from Carlo Erba Reagents. 3,5-Dimethylaniline, nitrobenzene, urea, ammonium molybdate, and 4-nitrophthalic anhydride were purchased from Fluorchem; concentrated hydrochloric acid 37%, phenol, NaNO_2_, NaOH, ZnCl_2_, and piperidine (99+%) were purchased from Carlo Erba Reagents; and malonodinitrile was purchased from TCI. Reactions were monitored by thin-layer chromatography (TLC) performed on alugram precoated silica sheets and 0.2 mm plates (MN), and compounds were visualized under UV light (254 nm or 365 nm), depending on the substrate. Column chromatography was performed on silica gel 60A (particle size 40–63 μm, Carlo Erba, Cornaredo, Italy) using positive air pressure.

(E)-4-((3,5-dimethylphenyl)diazenyl)phenol (AZO-OH): This compound was synthesized according to a published procedure [29]. Concentrated hydrochloric acid (2 mL) was added to a suspension of 3,5-dimethylaniline (0.606 g, 5.00 mmol) in water (20 mL). The mixture was cooled to 0 °C in an ice bath and stirred vigorously. A solution of NaNO_2_ (0.380 g, 5.50 mmol) in water (3 mL) was then added dropwise to the cooled suspension, and the mixture was stirred for 1 h. In a separate flask, NaOH 5 M (5 mL) was added to a solution of phenol (0.565 g, 6.00 mmol, 1.2 eq) in water (2 mL), and cooled to 0 °C. The former mixture was then slowly transferred to the solution of phenol and vigorously stirred for 1 h at 0 °C. The resultant precipitate was filtered in vacuo and purified by silica chromatography (dichloromethane/ethyl acetate 9.5:0.5) to give a yellow-orange powder (0.8 g, 72%). ^1^H NMR (600 MHz, CDCl_3_): δ = 7.86 (d, *J* = 8.8 Hz, 2H), 7.49 (s, 2H), 7.09 (s, 1H), 6.94 (d, *J* = 8.8 Hz, 2H), 5.08 (s, 1H), 2.41 (s, 6H). ^13^C NMR (151 MHz, CDCl_3_): δ = 158.2, 153.1, 147.5, 138.9, 132.3, 125.0, 120.5, 115.9, 21.5. The ^1^H and ^13^C NRM spectra are in good agreement with those published in the literature [35] (see S2 in Supplementary Materials).

[2,2′-bithiophene]-5-carbaldehyde: This compound was synthesized by adapting a procedure from the literature [36]. A Vilsmeier reagent was prepared by stirring POCl_3_ (0.56 mL, 6.02 mmol) and DMF (0.46 mL, 6.0 mmol) for 10 min and then adding them to an ice-cold solution of 2,2′-bithiophene (0.500 g, 3.01 mmol) in 1,2-dichloroethane (4 mL). The reaction mixture was heated to reflux for 24 h, hydrolyzed with saturated aqueous sodium acetate solution for 6 h, and extracted with dichloromethane (100 mL) three times. The organic layers were collected together, then dried over anhydrous sodium sulfate and filtered. The solvent was removed in vacuo to obtain the pure product as a yellow powder (0.580 g, 98%). The product was used in the next step without further purification. ^1^H NMR (600 Mhz, CDCl_3_) δ (ppm): 9.87 (s, 1 H), 7.67 (d, *J* = 3.9 Hz, 1 H), 7.36 (d, *J* = 4.4 Hz, 2 H), 7.25 (d, *J* = 3.9 Hz, 1 H), 7.08 (t, *J* = 4.4 Hz, 1 H). The ^1^H NMR spectrum is in good agreement with that published in the literature [37].

2-([2,2′-bithiophen]-5-ylmethylene)malononitrile (DCVBT): This compound was synthesized by adapting a published procedure [38]. To a solution of [2,2′-bithiophene]-5-carbaldehyde (0.200 g, 1.03 mmol) and malonitrile (0.057 mL, 1.03 mmol) in 20 mL of ethanol, under an inert atmosphere (Ar), 0.51 mL (0.515 mmol) of piperidine was added. The reaction mixture was refluxed (78 °C) for 4 h, and subsequently, it was allowed to reach room temperature. The solvent was removed via rotary evaporation, and the resulting solid was washed through solid–liquid extraction with diethyl ether (3 × 10 mL). The solvent was then removed using a pipette, and the process was repeated three times to obtain, finally, the desired product (0.204 g; 81.8%) as a red powdery solid. ^1^H NMR (600 MHz, CDCl_3_) δ (ppm): 7.77 (1H, s), 7.64 (1H, d, *J* = 4.1 Hz), 7.43 (2H, d, *J* = 4.1 Hz), 7.28 (1H, d, *J* = 4.2 Hz), 7.11 (1H, t, *J* = 4.2 Hz). ^13^C NMR (151 MHz, CDCl_3_) δ: 150.2, 149.3, 139.9, 135.2, 133.5, 128.7, 128.3, 127.2, 124.6, 114.1, 113.3 (see S2 in Supplementary Materials).

Tetra-amino-zinc-phthalocyanine (PcZnNH_2_): This compound was synthesized according to published procedures. Ammonium molybdate (6.5 mg, 0.005 mmol) was added to a solution of 4-nitrophthalic anhydride (1.0 g, 5 mmol), urea (1.5 g, 25 mmol), and zinc chloride (190 mg, 1.3 mmol) in nitrobenzene (7.5 mL). The mixture was stirred under N_2_ at 185 °C. After 4 h, the reaction mixture was cooled and diluted with toluene (40 mL). The resulting precipitate was collected by centrifugation. The solid was washed with toluene, water, MeOH/ether (1:9), and EtOAc/hexane (2:1), and dried to afford a dark green solid (0.6 g, 62%). The recovered product was used without further purification for the next step. Sodium sulfide nonahydrate (2.3 g, 9.5 mmol) was added to a solution of tetranitro-zinc-phthalocyanine (0.6 g, 0.8 mmol) in DMF (16 mL). The reaction mixture was stirred under N_2_ and heated at 60 °C. After 1.5 h, the mixture was cooled to room temperature and diluted with ice water (50 mL), and the resulting precipitate was collected by centrifugation. The precipitate was repeatedly washed with MeOH/ether (1:9) and EtOAc and dried to afford a dark green solid (0.4 g, 79%). ^1^H-NMR (600 MHz, d_6_-DMSO) δ: 8.12–8.03 (m, 4H), 7.58 (d, *J* = 23.1 Hz, 4H), 6.52 (s, 4H), 5.36 (s, NH_2_).

^13^C NMR (151 MHz, DMSO) δ: 162.3, 154.9, 154.2, 153.9, 153.7, 153.7, 153.2, 153.1, 153.0, 152.8, 152.6, 152.5, 152.1, 151.90, 151.88, 151.38, 151.35, 151.3, 151.1, 151.1, 151.0, 150.8, 150.7, 140.9, 140.81, 140.76, 140.6, 140.5, 140.4, 140.3, 126.9, 126.80, 126.77, 126.75, 126.70, 126.6, 123.33, 123.27, 123.2, 123.1, 123.0, 122.9, 122.9, 122.8, 116.03, 116.00, 115.96, 105.8, 105.7, 105.6, 105.5, 105., 105.34, 105.16, 105.12.

The ^1^H and ^13^C NMR spectra are in good agreement with those published in the literature [39] (see S2 in Supplementary Materials).

### 3.2. NMR

^1^H and ^13^C liquid NMR spectra were recorded on a Bruker Advance III HD 600 MHz NMR spectrometer at 298 K. Deuterated chloroform was used to prepare the NMR sample and was purchased from Carlo Erba Reagents. Chemical shifts (δH and δC) are expressed in parts per million (ppm) relative to the residual solvent peak and, for proton NMR, shown as follows: chemical shift, multiplicity (s = singlet, d = doublet, t = triplet, q = quartet, m = multiplet), coupling constant (*J*, Hz), and integration.

### 3.3. Raman

Micro-Raman spectra were obtained from solid molecules through the Near infrared micro Raman B&W-TEK (Newark, NJ, USA) i-Raman Ex integrated system in back scattering geometry with the 1064 nm line of an Nd:YAG laser. The experimental setup, equipped with the Video Micro Sampling System BAC151B and a 20× Olympus objective, guarantees a spectral resolution of 8 cm^−1^.

### 3.4. Steady-State Absorption, Photoluminescence Excitation (PLE), and Quantum Yield

UV-Vis-NIR solid-state absorbance spectra were collected (applying baseline corrections) by a Jasco V-750 spectrophotometer with a spectral bandwidth of 2 nm in the 200–900 nm range.

Three-dimensional fluorescence mapping of samples was performed using a spectrofluorometer equipped with an integrating sphere, a Jasco FP-8050, with a 450 W xenon lamp as the excitation source. The maps were collected with an excitation range of 200–600 nm and an emission range of 250–850 nm with a 5 nm spectral bandwidth for excitation and emission.

### 3.5. Time-Resolved Photoluminescence (TRPL)

For time-resolved luminescence measurements, an optical parametric oscillator with a frequency doubler device was utilized, driven by the third harmonic (355 nm) of a pulsed Nd:YAG laser (Quanta Ray Pro 730). The excitation pulse had a width at half-maximum of 8 ns, a repetition rate of 10 Hz, and a spectral bandwidth of less than 0.3 cm^−1^. Signals were captured using a spectrograph (Arc-SpectraPro 300i) with a spectral bandpass of less than 2.5 nm and detected by a gateable intensified CCD (PI MAX Princeton Inst.). To minimize dark current, the detector was cooled to −20 °C using a Peltier device.

### 3.6. Transient Absorption (TA)

For TA measurements, a regenerative Ti:Sapphire amplifier Coherent Libra-F-1K-HE230 produced a train of laser pulses with 100 fs at 800 nm and a 1 kHz repetition rate. The train of laser pulses is divided into two parts by a beam splitter, called the pump and probe, respectively. The pump laser pulse is sent on an optical parametric amplifier (TOPAS-800-fs-UV-1) and finally focused not perpendicularly on the sample after a chopper (500 Hz) is synchronized with the frequency of the source. A white super-continuum pulse (probe) is formed by multifrequency generated by a sapphire plate which also guarantees sufficient stability and bandwidth flatness. The probe pulse, after passing a controlled delay line, is focused and sent on the sample in the same intersection area of the pump pulse. After the interaction with the sample, the transmission signal is collected into the detection system (Ultrafast Systems HELIOS-80000-UV-VIS-NIR coupled with a CCD camera). For every single step of the delay line, a single-wavelength dispersed differential transmission spectrum is acquired, obtaining a time spectrogram that can provide information about the temporal dynamics of the energy level depletion.

### 3.7. DFT Calculations

All the quantum chemistry calculations were performed using the Gaussian 16 suite of programs. We performed geometry optimization down to the self-consistent field (SCF) energy of the monomer by means of DFT calculations carried out at a B3LYP/6311++G(d,p) [40] level of theory. In the case of aggregates, the ωB97XD/6–311++G(d,p) [41] level of theory was applied, as already reported in [42]. The interaction of molecules with solvents was accounted for by applying the self-consistent reaction field (SCRF) model and simulating the dielectric solvent through the Polarizable Continuum Model (PCM) calculation within the integral equation formalism (IEFPCM) [43]. All the structures were verified to be real energy minima with no imaginary frequencies in the vibrational spectra.

All the UV absorption spectra were computed at the B3LYP/6311++G(d,p) level of theory. The spectra were simulated as vertical energy transitions with the solvent environment assumed to be the same as the ground state of the molecule.

Regarding the computed Raman features, we applied a quadratic scaling factor [44].

### 3.8. Docking Calculations

Ensemble-docking calculations were performed using the software GNINA [28]. The conformations of α-syn (clusters C1 to C8) were taken from ref. [31]. Three-dimensional molecular models of the molecules were generated from 2D molecular sketches using MarvinSketch (version 14.9.1.0, calculation module developed by ChemAxon, http://www.chemaxon.com/marvin, accessed on 9 October 2023) and further optimized with the Gaussian 16 suite of programs [Gaussian] in the presence of implicit water solvent [keyword: #b3lyp/6-31G(d,p) opt(tight) scf(MaxCycle = 512) scrf = (pcm, solvent = water) freq].

Considering the intrinsically high conformational variability of the protein, blind docking calculations were performed on each of the eight structures whilst increasing the exhaustiveness so as to improve sampling (keywords: autobox_ligand = C_X.pdb—where X is the cluster number; autobox_add = 8; autobox_extend = 1; scoring = default; exhaustiveness = 4096; num_modes = 100).

Docking poses were ranked according to the estimated affinity of binding and clustered using the cpptraj module of AMBER22 (D.A. Case, H.M. Aktulga, K. Belfon, I.Y. Ben-Shalom, J.T. Berryman, S.R. Brozell, D.S. Cerutti, T.E. Cheatham, III, G.A. Cisneros, V.W.D. Cruzeiro, T.A. Darden, R.E. Duke, G. Giambasu, M.K. Gilson, H. Gohlke, A.W. Goetz, R. Harris, S. Izadi, S.A. Izmailov, K. Kasavajhala, M.C. Kaymak, E. King, A. Kovalenko, T. Kurtzman, T.S. Lee, S. LeGrand, P. Li, C. Lin, J. Liu, T. Luchko, R. Luo, M. Machado, V. Man, M. Manathunga, K.M. Merz, Y. Miao, O. Mikhailovskii, G. Monard, H. Nguyen, K.A. O’Hearn, A. Onufriev, F. Pan, S. Pantano, R. Qi, A. Rahnamoun, D.R. Roe, A. Roitberg, C. Sagui, S. Schott-Verdugo, A. Shajan, J. Shen, C.L. Simmerling, N.R. Skrynnikov, J. Smith, J. Swails, R.C. Walker, J Wang, J. Wang, H. Wei, R.M. Wolf, X. Wu, Y. Xiong, Y. Xue, D.M. York, S. Zhao, and P.A. Kollman (2022), Amber 2022, University of California, San Francisco.) in order to identify non-overlapping poses on the whole surface of each protein conformation. For this purpose, we chose an RMSD cutoff of 5 Å.

## 4. Conclusions

In this work, we investigated three molecular scaffolds as possible probes for the early detection of Parkinson’s disease. We found that all the candidate compounds are able to bind aggregation-prone conformations of α-synuclein, particularly at the β-sheet motifs that are crucial to the onset of pathogenic pathways (Figure 3 and S3 in Supplementary Materials) [45]. Moreover, we demonstrate in Section 2.5 that among these molecules, DCVBT and PcZnNH_2_ exhibit good absorption properties, high luminescence, and good 2PE properties as expected, features that promote them as possible candidates for biomedical applications. Finally, DCVBT was also deeply investigated in water solvent to account for possible applications in biomedical fields. The optical features were interpreted based on the insights gathered by quantum chemistry calculations, showing that the formation of aggregates opens new excitation channels in the visible range. Thus, collecting all the reported properties and considering its composition, small molecular weight, and reduced dimensions, DCVBT appears as the most promising candidate among the analyzed set.

## Figures and Tables

**Table 1:** Affinities of the top clusters to α-syn for three molecules (kcal/mol), as estimated from the docking scoring function of GNINA.

Cluster *	DCVBT	AZO-OH	PcZnNH_2_
C1	−4.6	−5.7	−9.3
C3	−4.7	−6.6	−9.4
C5	−4.9	−5.3	−9.1
C6	−4.7	−5.9	−9.3

The “*” refers to reference [31].

## Data Availability

Data are contained within the article and Supplementary Materials.

## Supplementary Materials

The following supporting information can be downloaded at https://www.mdpi.com/article/10.3390/molecules29122817/s1: Scheme S1: synthetic schemes; S1: Raman vibrational modes; S2: NMR spectra; S3: Distribution of docking poses of the three molecules investigated in this work on the surface of a-syn. The centers of mass of each pose are shown as spheres of size and color proportional to the docking score (in kcal/mol in the color bars accompanying each picture). The protein is shown using the cartoon representation and colored by secondary structure; S4: HOMO-LUMO orbitals for DCVBT, AZO-OH, PcZnNH2. The isocontour value is 0.02 au; S5: Two-Photon emission of DCVBT under excitation at 840 nm and 900 nm.
