# Supplementary material for: Promising Molecular Architectures for Two-Photon Probes in the Diagnosis of α-Synuclein Aggregates
Source: Molecules. 2024 Jun 13;29(12):2817. doi: 10.3390/molecules29122817 (PMC11207056; doi:10.3390/molecules29122817)
Supplement: Supplementary file 1 [file molecules-29-02817-s001.zip › molecules-2995466-supplementary.pdf]

Supplementary Materials

Synthetic Schemes

a)

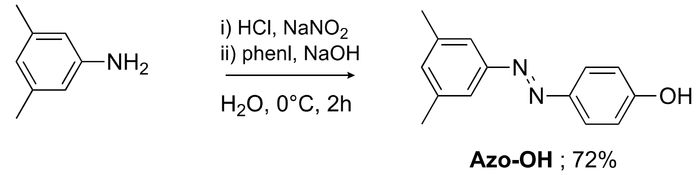

b)

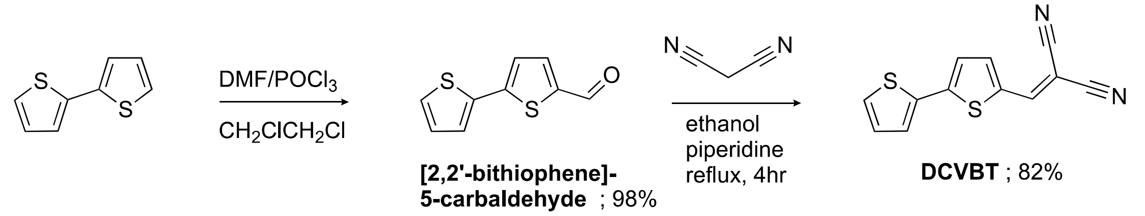

c)

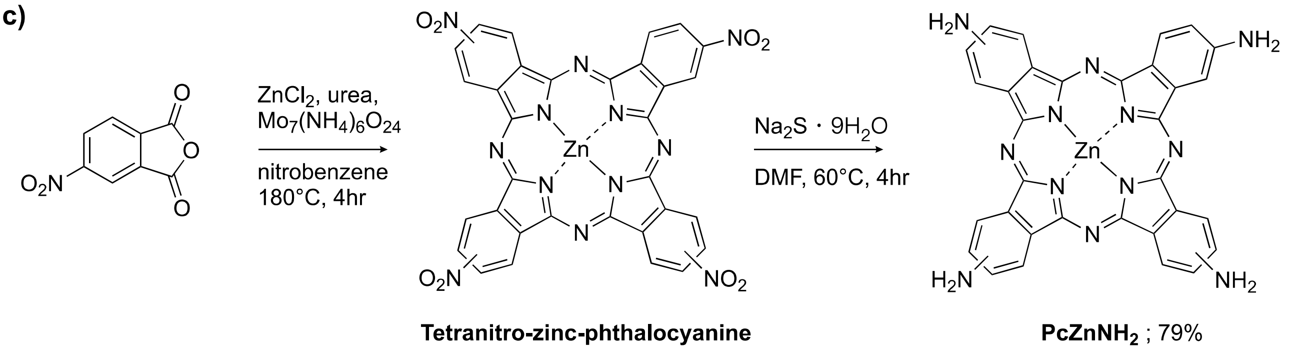

Scheme S1: Synthetic path the of the investigated compounds: **Azo-OH** (a); **DCVTBT** (b); **PcZnNH₂** (c)

S1. Raman vibrational modes.

DCVBT

**Vibrational Mode**	**Frequency (cm^{-1})**
ν(C≡N) of cyano bonds	2220
Symmetric stretching νs(C=C)	1567
Antisymmetric stretching νa(C=C)	1525
C=C/C-C motions	1432, 1414
Bending δ(C-C)	1336
Stretching of C-C bonds	1235, 1193
Internal bending vibrations δ(C-H)	1140, 1062
Relative motions among macro structures (e.g., thiophenes or cyano groups)	Below 750cm^{-1}

AZO-OH

**Vibrational Mode**	**Frequency (cm^{-1})**
Symmetric stretching ν of the ring	1600, 1477, 1430
In-plane bending β for rings' CH	1313, 1268, 1211
Symmetric stretching ν for CO	1286, 1133, 1026, 993
Symmetric stretching ν for vinylidene CC	1154
Typical vibration modes under for out-of-plane bending for CH (ring), CC, and CO bonds	Below 800 cm^{-1}

PcZnNH2

**Vibrational Mode**	**Frequency (cm^{-1})**
A1g modes	1413, 1317, 830, 687
E modes	1600, 1261, 1044, 781, 745
B2g modes	1603, 1115
Relative motions among macro structures	Below 600 cm^{-1}

S2. NMR Spectra

^{1}H NMR Compound AZO-OH

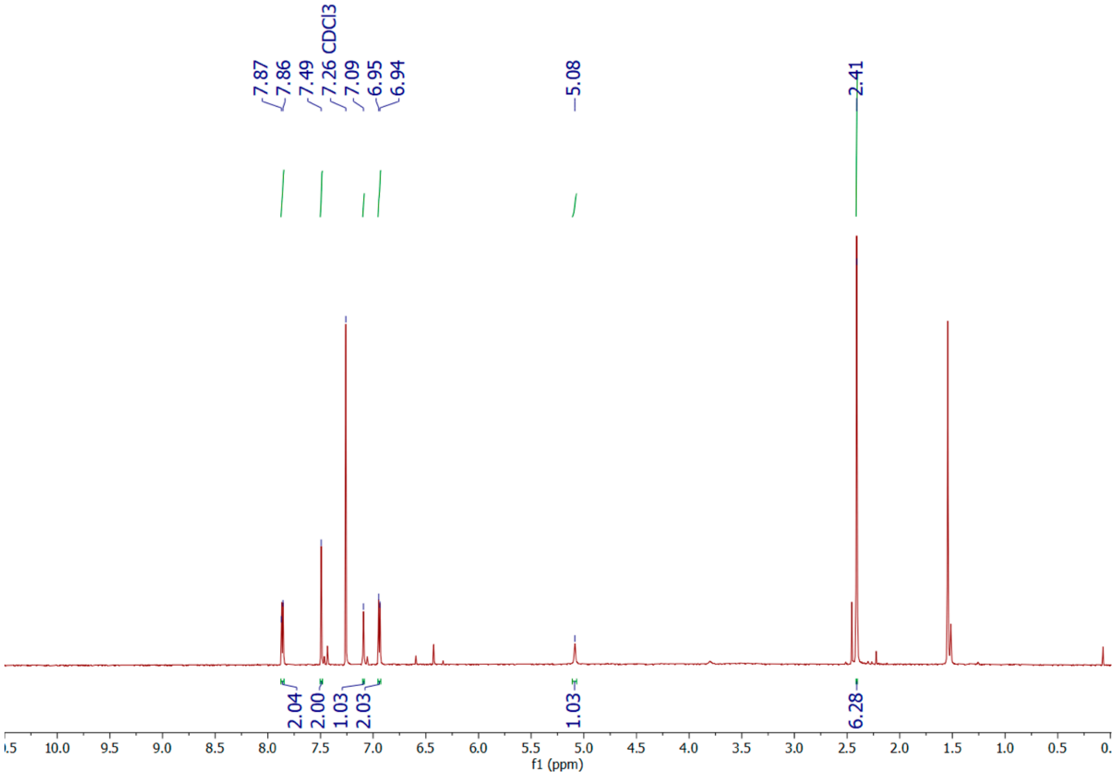

^{1}H NMR Compound DCVBT

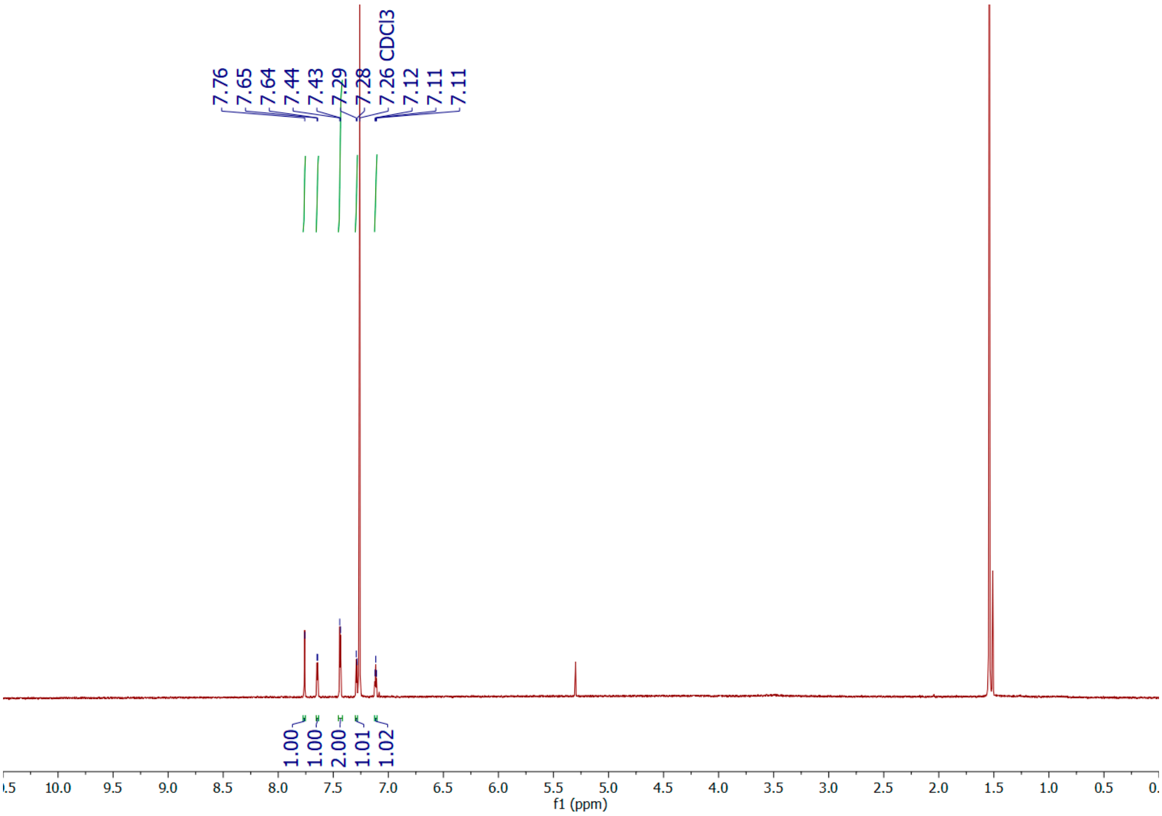

^{1}H NMR Compound $PcZnNH_2$

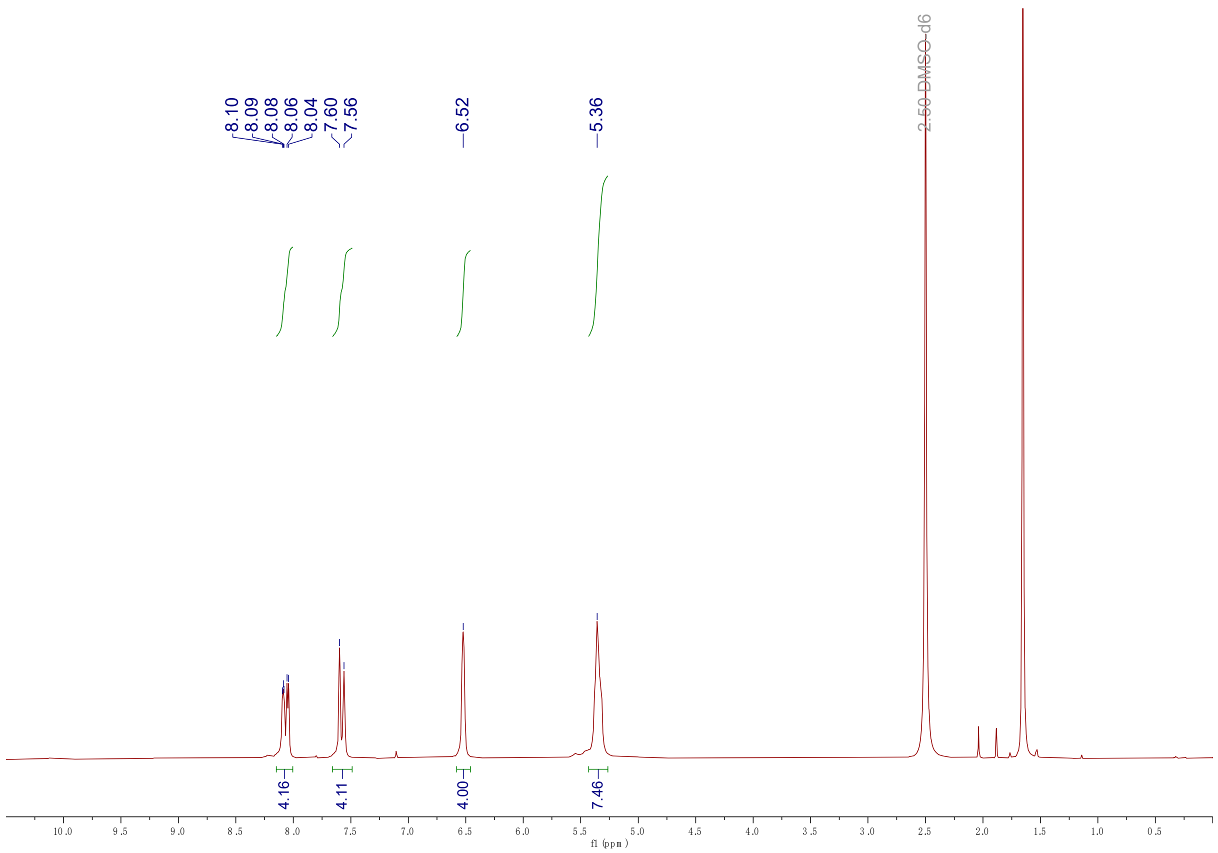

^{13}C NMR Compound AZO-OH

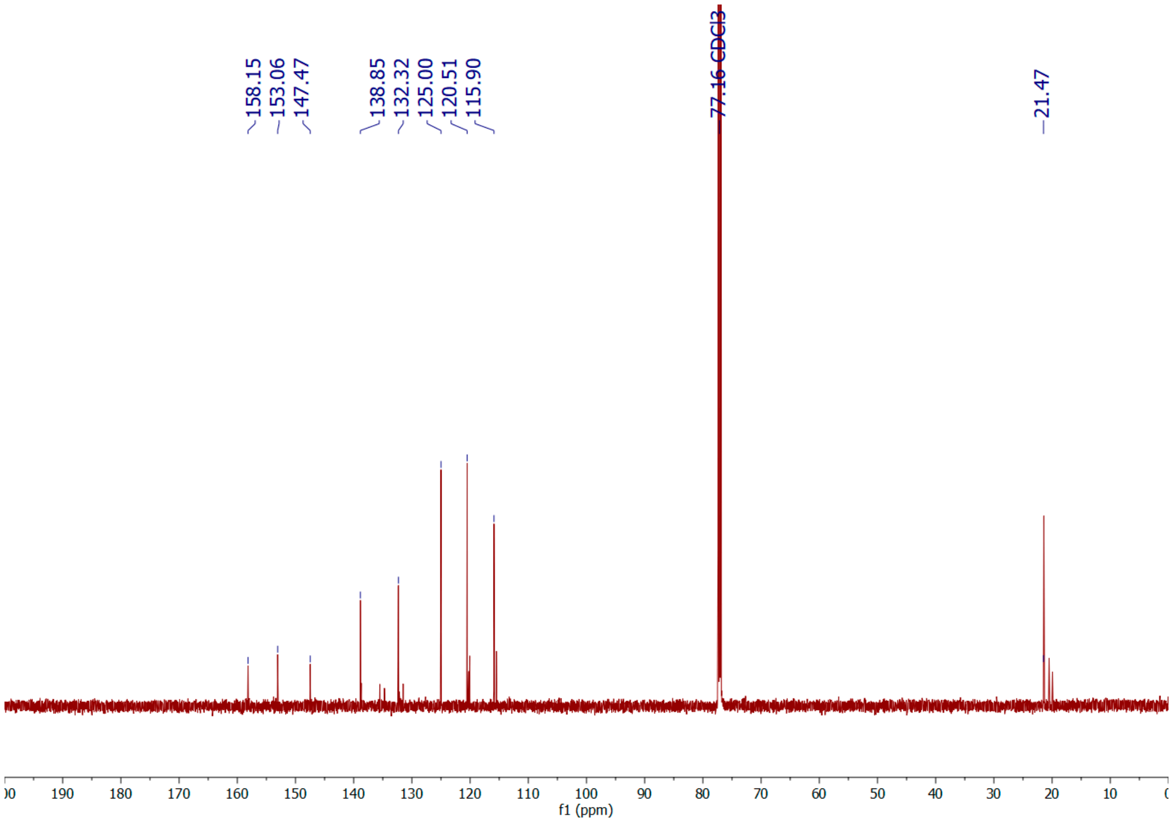

^{13}C NMR Compound DCVBT

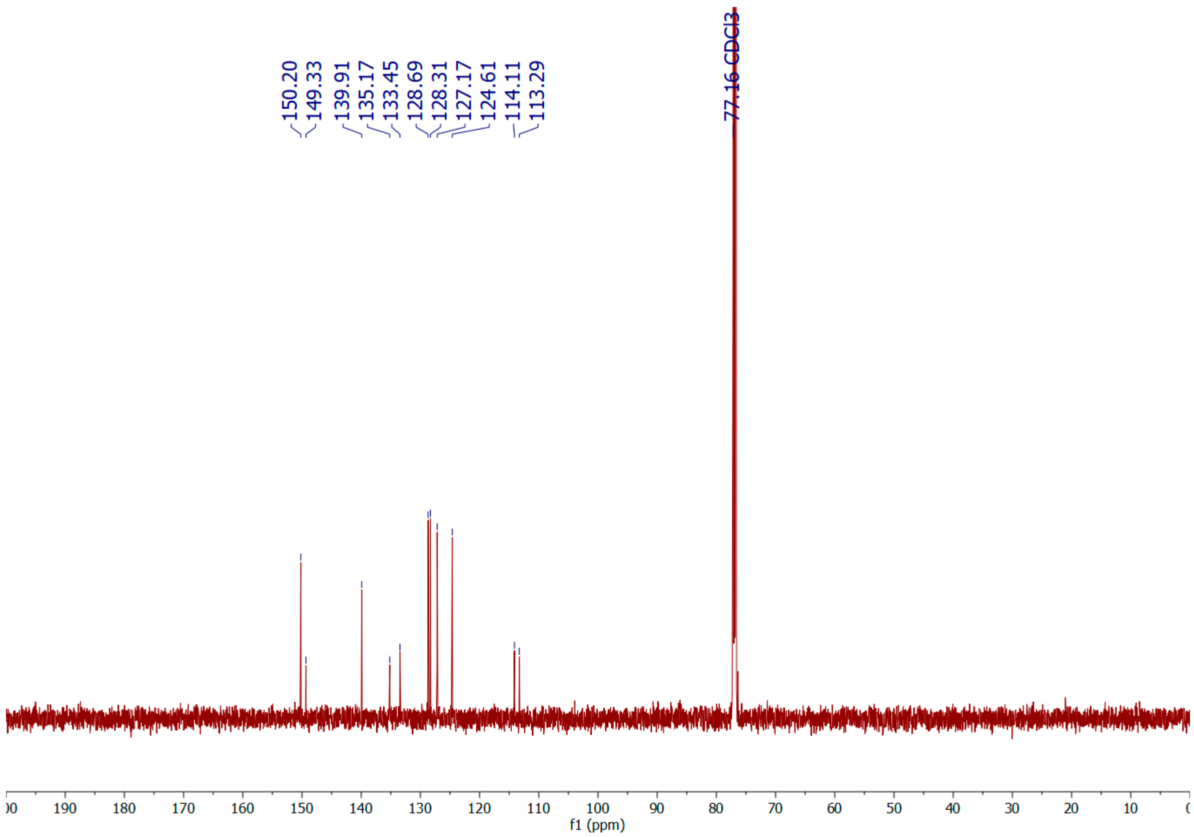

^{13}C NMR Compound PcZnNH2

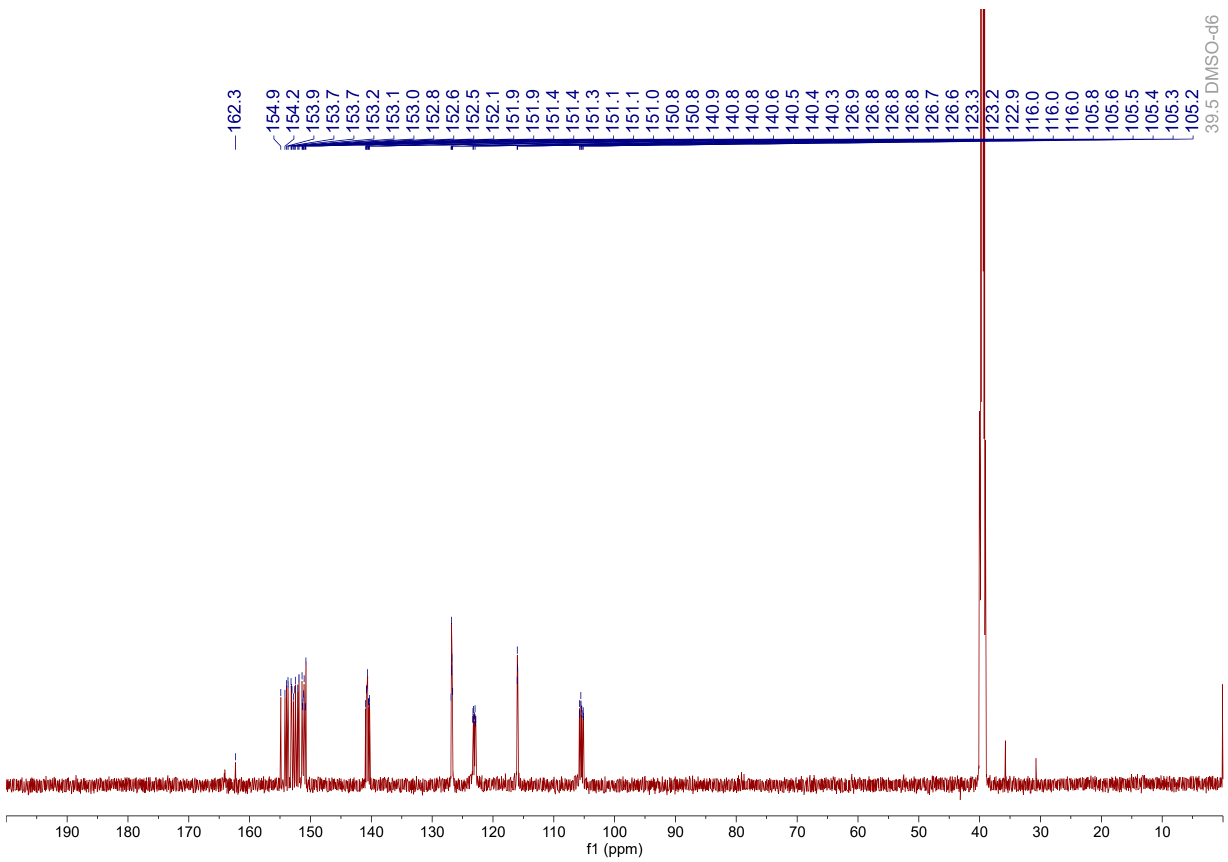

S3. Distribution of docking poses of the three molecules investigated in this work on the surface of a-syn. The centers of mass of each pose are shown as spheres of size and color proportional to the docking score (in kcal/mol in the color bars accompanying each picture). The protein is shown using the cartoon representation and colored by secondary structure.[1]

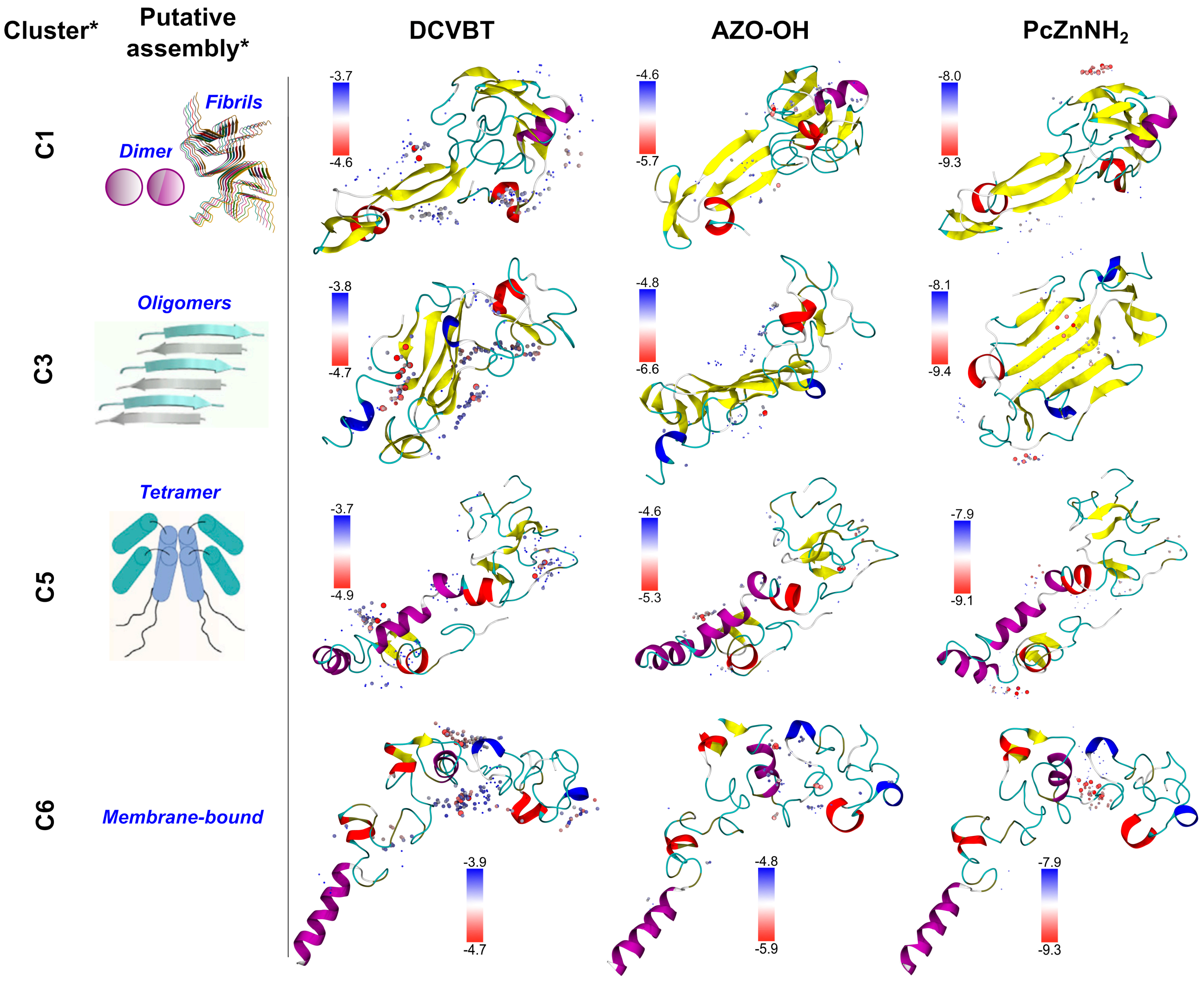

S4. HOMO-LUMO orbitals for DCVBT, AZO-OH, $PcZnNH_2$. The isocontour value is 0.02 au.

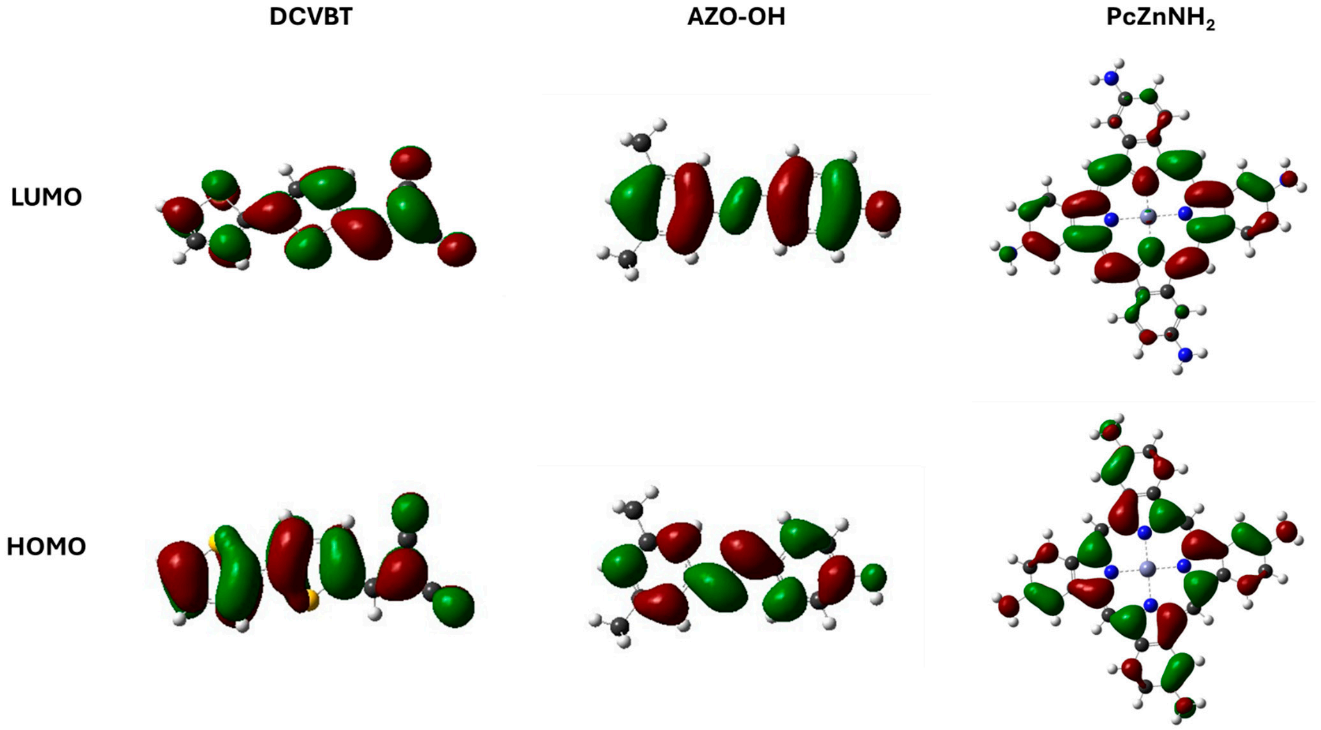

S5. Two-Photon emission of DCVBT under excitation at 840 nm and 900 nm

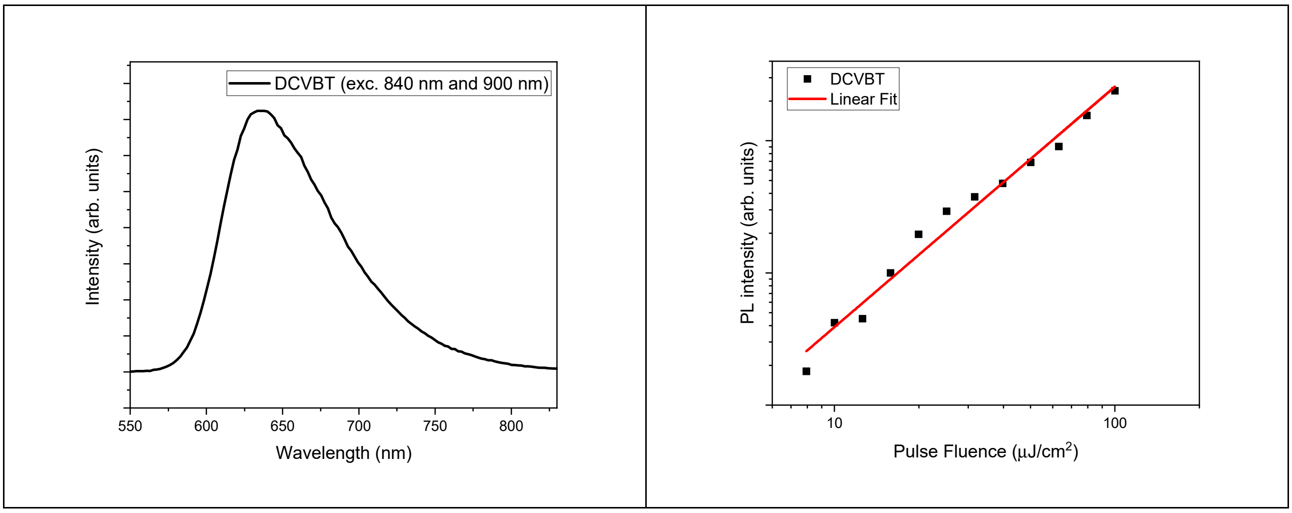

References

[1] J. Chen, S. Zaer, P. Drori, J. Zamel, K. Joron, N. Kalisman, E. Lerner, N. V. Dokholyan, Structure 29 (2021) 1048-1064.e6.
